# Effectiveness of an inexpensive short-term theoretical-practical course on videosurgery for surgeons in training

**DOI:** 10.1186/s12909-022-03594-2

**Published:** 2022-07-07

**Authors:** Paula Haveroth Takegawa, Jefferson Kalil, Joaquim Murray Bustorff-Silva, Márcio Lopes Miranda

**Affiliations:** Division of Pediatric Surgery, Department of Surgery, Faculty of Medical Sciences, State University of Campinas, Rua Tessalia Vieira de Camargo 126, Campinas, SP 13083-887 Brazil

**Keywords:** Laparoscopic video surgery, Medical residency, Medical education, Technical abilities

## Abstract

**Background:**

The rapid development of video surgery and minimally invasive surgical techniques prompted many studies on the methods of teaching these techniques to young surgeons in training. However, the characteristics of a short-term course that is both easily accessible and efficient for this group of surgeons remain controversial. To investigate this issue, a short-term training method was proposed for first year surgery residents, using inexpensive handmade wooden simulation boxes with the students smartphones as cameras. Its effectiveness was evaluated, as well as possible factors that could influence student performance, such as gender and previous experience with video games.

**Methods:**

Thirty-six first-year General Surgery residents, entering in 2019 and 2020, participated in the study: 21 were males and 15 were females with ages between 22 and 29 years old, (mean 25.47 years). All participants performed a pre-established exercise (placing two simple stitches using a laparoscopic simulator), which was timed and scored. They then participated in a short theoretical-practical course, consisting of an initial lecture followed by 4 exercises on handcrafted wooden laparoscopic video surgery simulators. Afterwards, they were asked to repeat the same exercise from the first step. Finally, they answered a questionnaire that included questions on previous videogame experience. The data were tabulated and submitted to statistical analysis.

**Results:**

In the pre-training exercise, 15 (41.66%) participants were able to perform the two simple stitches in the simulator box within the maximum time limit of 5 minutes. After the short course, 22 (61.11%) of participants were able to perform the complete exercise. Improvement in the time to complete the practical exercise was statistically significant (*p* = 0.0296) after participating in the theoretical-practical course. A better pre- and post-training performance was demonstrated by the 17 participants with experience with video games (*p* = 0.0116), and a better post-training performance was demonstrated by female participants (*p* = 0.0405).

**Conclusion:**

This short-term inexpensive theoretical-practical course in laparoscopic training for surgeons in training was effective in reducing the execution time of a laparoscopic stitch in a simulation box. Previous experience with video games and/or female gender appear to be associated with improved performance.

## Background

In recent years, several studies have been published on the subject of teaching of video-assisted surgery, aiming to improve the learning curve for the current surgical techniques, and to develop more appropriate and effective practical methods to train new surgeons [1–14]. Present methods range from the use of handcrafted boxes to sophisticated virtual reality simulators; all aimed at developing new surgical skills, avoiding the ethical aspects of in vivo teaching practices, minimizing potentially errors and reducing the cost of surgeons’ training [13, 15, 16].

There are evidences that assisted training, with an instructor guiding the residents across the activities, is superior to self-taught training during General Surgery residency. The teachers’ perceptions and feedback about individual abilities and limitations are considered essential for the residents’ proper development [13, 17]. However, the creation of simplified methodologies, which can be accomplished outside the hospital environment, may accelerate the learning process, allowing to improve on the lessons learned in a supervised manner in a surgical technique laboratory [12, 17].

One of the main reasons for instituting a training course in the beginning of the residency program, was to introduce the residents to video assisted procedures early in the beginning of surgical training. Also, differently from other studies that evaluate undergraduate students, we wanted to evaluate the method in a group of residents who have already defined an interest in surgery, but don’t have any practical knowledge in video-assisted techniques yet.

### Objective

The main objective of this study was to evaluate the effectiveness of a short-term practical training course, using inexpensive video surgery simulators, in the development of students’ surgical skills at the start of their surgical training.

As a secondary goal, we sought to investigate the possible influence of other factors (gender and regular use of video games) on the performance of the participants.

## Methods

The study was approved by the Research Ethics Committee of the State University of Campinas (CEP Unicamp), CAAE number 20090419.4.0000.5404.

The required sample size was calculated based on a hypothetical post-training score increase of 1 point, considering 1.5 points as the standard deviation. Considering α = 0.05 and 1-β = 0.95, the required sample size was calculated to be 27 individuals. As 18 residents are admitted to the Department of Surgery each year, it was decided to evaluate residents for two consecutive years. Thus, all residents entering the General Surgery Residency Program at the State University of Campinas Medical School in 2019 and 2020 were included, totalling 36 participants. None of the participants had any previous formal or informal training in laparoscopic skills.

In order to prevent any bias in the behaviour of teachers and evaluators in the 2020 group, neither the score results, nor the questionnaires for 2019 were opened or analysed by any of the authors, until the 2020 residents’ assessment was completed.

Of the 36 participants, 21 were male and 15 were female, with ages ranging from 22 to 29 years (mean = 25.47 years).

The short-term theoretical-practical course on laparoscopic surgery consisted of five steps:

Stage 1: 40-minute lecture, explaining the basic skills and concepts of video-assisted surgery and detailing the training techniques in simulators. At the end of the lecture, the research project was fully explained and the participants were asked to sign the informed consent.

**Fig. 1:**
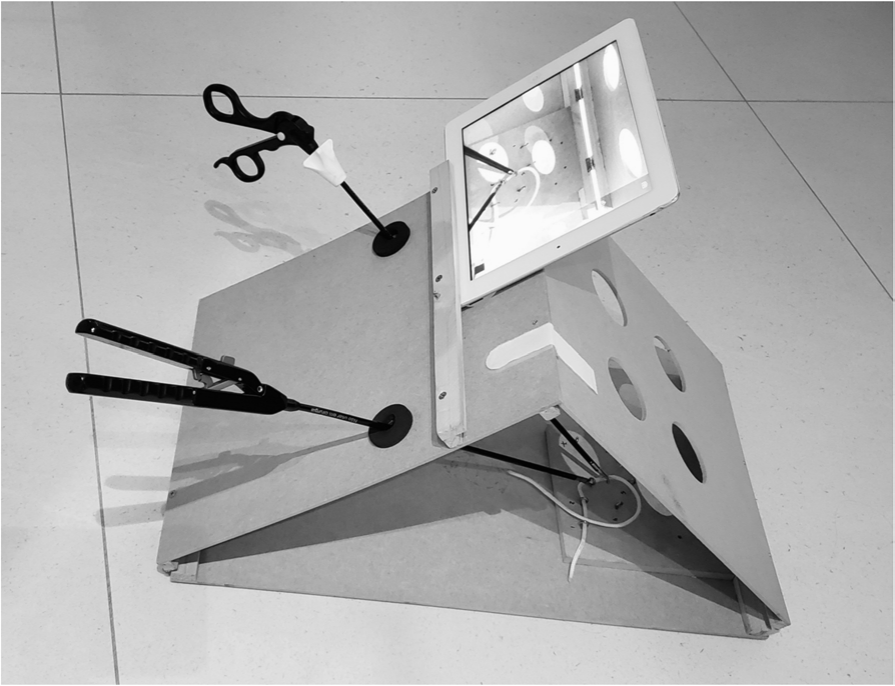
Simulation box made with simple, unexpensive, readily-available materials, using the students own smartphone or tablet as a camera

Stage 2 - (pre-training test): application of a timed practical test in the laparoscopic surgery simulator box consisting of two simple suture stitches in a sample of animal tissue. The exercise was performed in a simulator box (Lapbox), using an Olympus video monitor and a Storz image generator. A time limit of 5 minutes to complete the stitch was established a priori. The exercise was performed in pairs, with both residents alternating between recording the exercise performed by their colleague, and their own performance of the laparoscopic exercise;

Stage 3: supervised practical training with each exercise divided into quick steps (20 minutes). Instructions were offered for skill development with video surgery instruments and improvement in spatial vision of the two-dimensional camera by using handcrafted simulation boxes made with low-cost materials and the participants’ own cellular phone or tablet (Fig. 1).

Stage 3 exercises were developed in the following order:

1. Insertion of latex rings into a cut latex glove finger (20 minutes);
2. Fitting of latex rings to a vertical pin (20 minutes) using both hands;
3. Passage of needled thread between fixed rings (20 minutes), via the transfer and
4. Placing two simple stitches with 2–0 sutures in a silicone piece for (40 minutes). Emphasis on positioning the needle, passing the needle, and positioning the suture thread.;

Stage 4 (post-training): re-application of the timed practical test in the video surgery simulator box.

Stage 5: conclusion of practical training activities and application of a short questionnaire to evaluate previous experience with video surgery, any video game habits and level of satisfaction with the course. Additionally, at the end of the course, participants were given a tutorial on how to produce their own laparoscopic training boxes with easy-to-find materials, encouraging future independent practice.

For statistical analysis, participants’ times were converted into a score from 0 to 6 as shown in the table below (Table 1):

**Table 1:** Scoring table for pre- and post-training test, according to the time taken to complete the exercise

Time interval	Score
< 1 min	6
≥1 min and < 2 min	5
≥2 min and < 3 min	4
≥3 min and < 4 min	3
≥4 min and < 5 min	2
≥5 min (not completed)	1

Data was tabulated and analyzed using the GraphPad Prism 9.0 version. Pre- and post-training scores were compared using the Wilcoxon test for paired samples. To analyze the association between participants’ previous experience with video games and skills demonstrated in video surgery, Mann-Whitney’s non-parametric test was used. Differences in performance between female and male participants were compared using Kruskal-Wallis’ non-parametric test.

## Results

In the pre-training exercise, 15 (41.66%) participants were able to complete the two simple stitches in the simulator box within the maximum time limit of 5 minutes. After completing the course, 22 (61.11%) participants were able to perform the complete exercise (*p* > 0.05). This difference did not reach statistical significance.

**Fig. 2:**
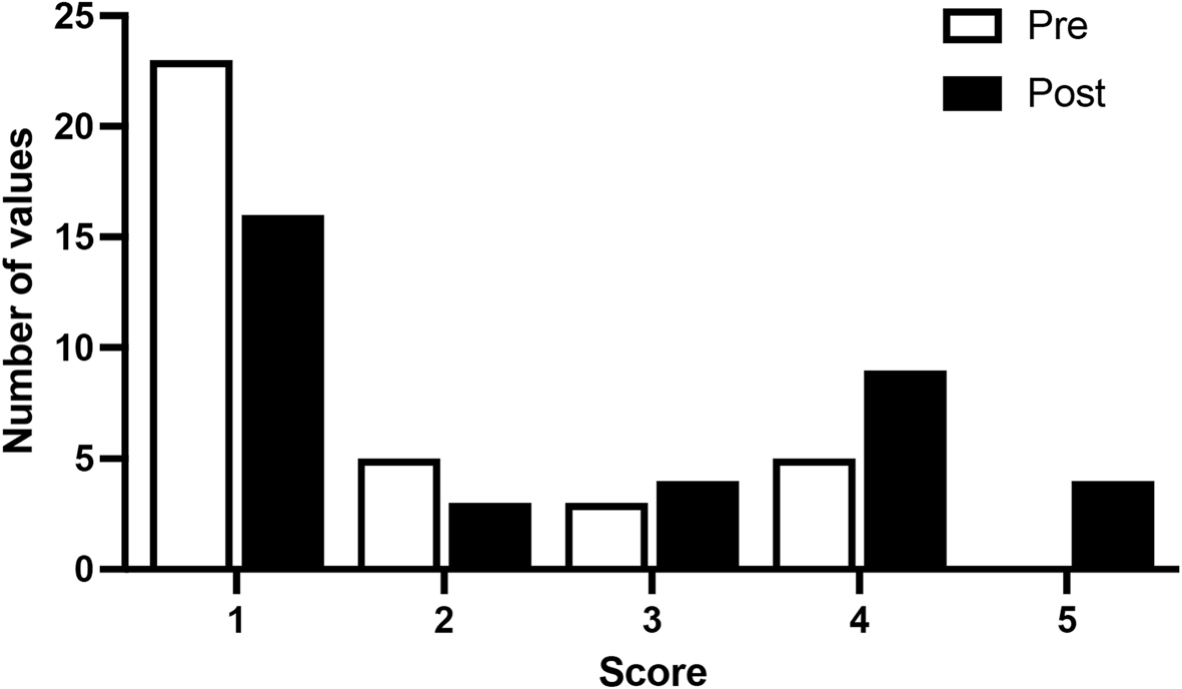
Graphical representation of the distribution of individual pre- and post-course scores

**Fig. 3:**
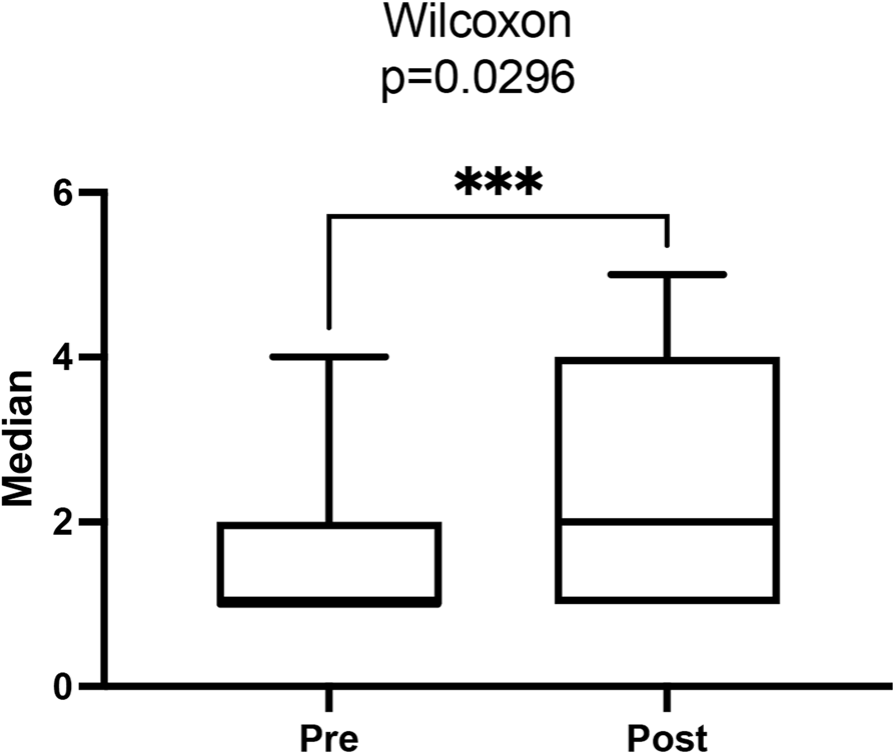
Graphical representation of the median of pre- and post-course scores

However, analysis of the data in Figs. 2 and 3 shows a significant improvement in the median scores obtained in the evaluations of pre- and post-training tests (*p* = 0.0296), reflecting the decrease in time spent performing the task assigned.

**Fig. 4:**
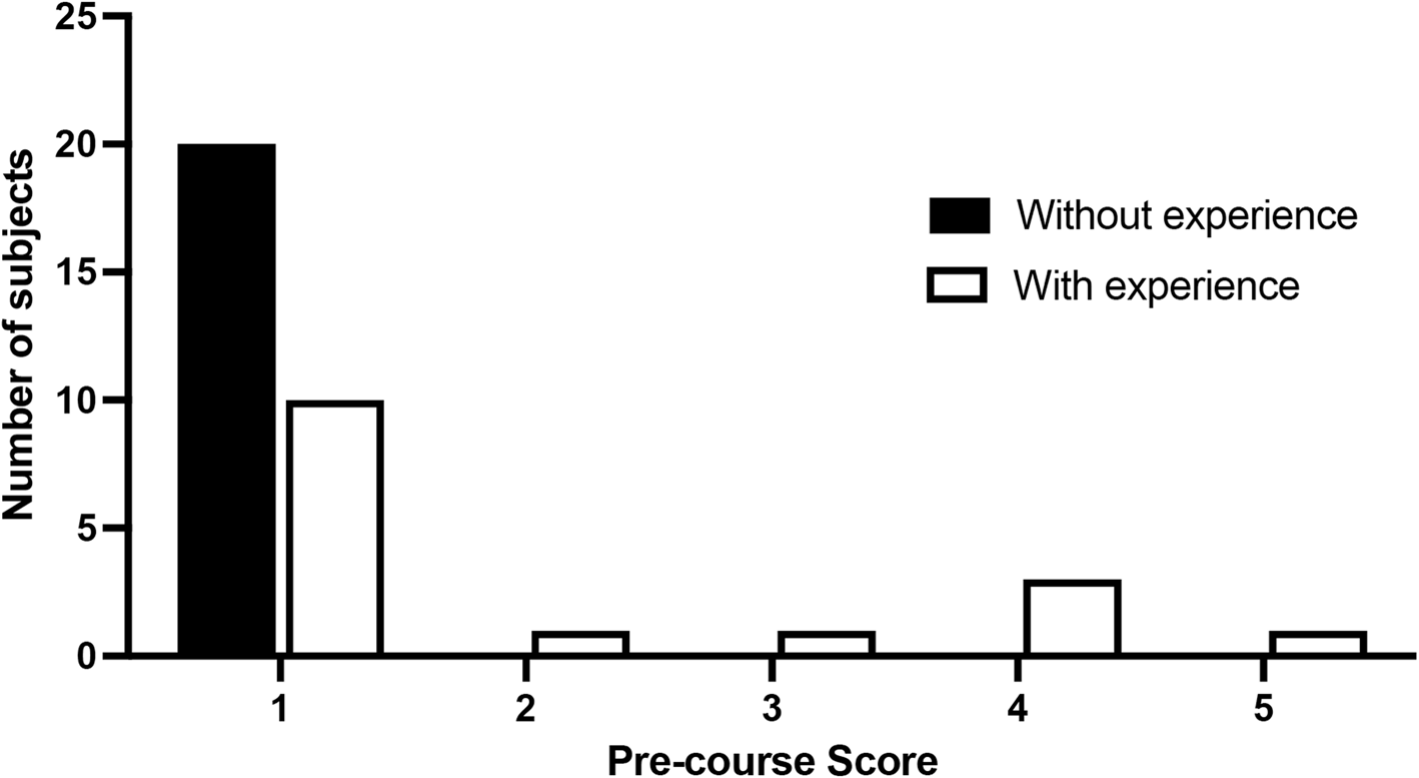
Graphical representation of the distribution of scores among residents with or without videogame experience

**Fig. 5:**
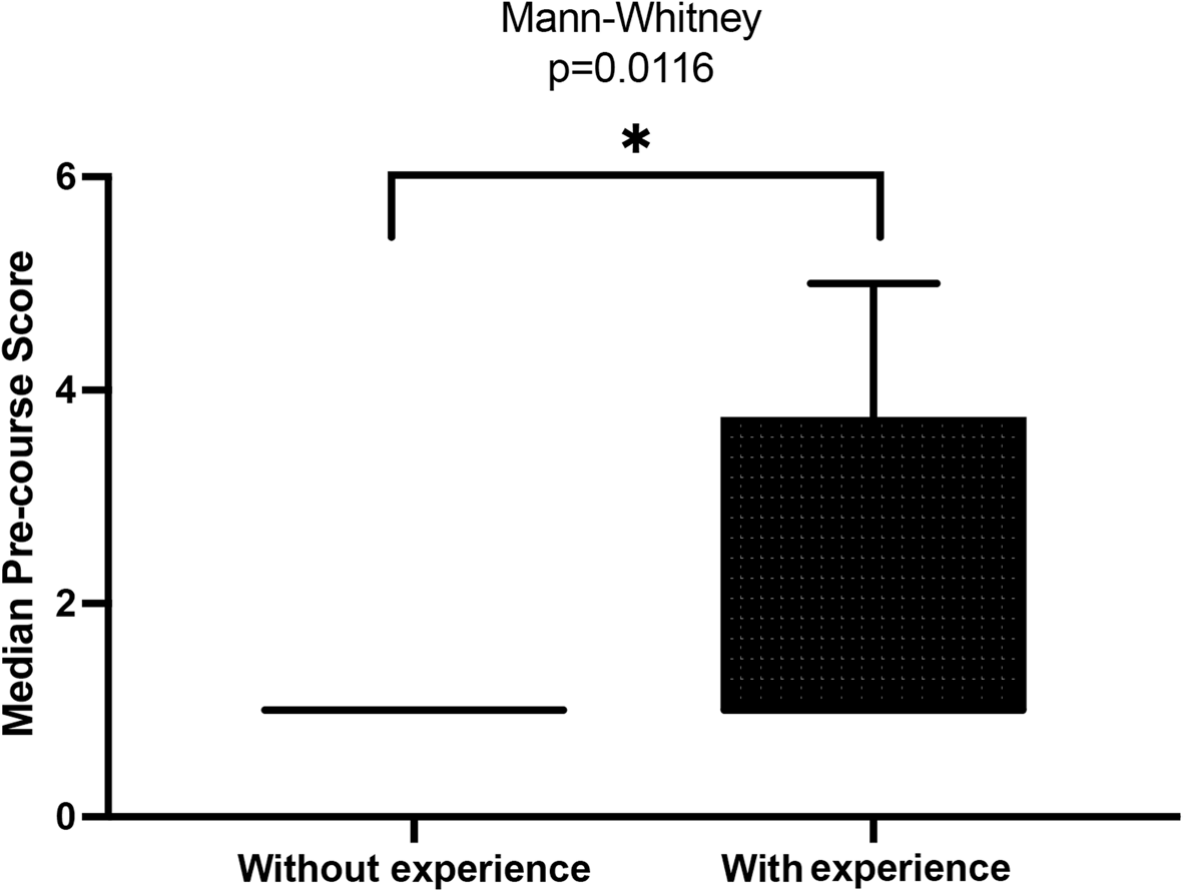
Graphical representation of the pre-course scores comparing residents with or without videogame experience

Regarding the impact of previous experience with video games, 17 (47.22%) had previous experience with video games and 19 (52.77%) had none. There was a significant difference in the pre-training scores of individuals with prior experience compared to the ones without (*p* = 0.0116; Figs. 4 and 5).

**Fig. 6:**
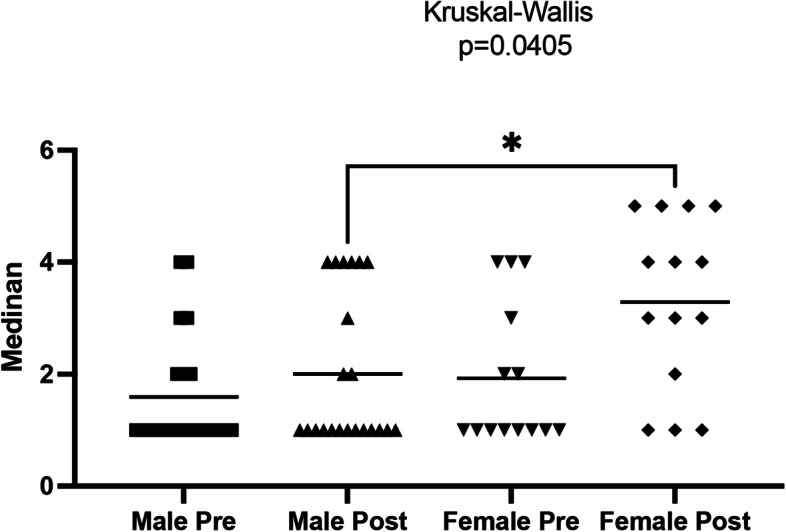
Graphical representation of the individual pre- and post-course scores classified by participant’s gender

Data from Fig. 5 shows that the female residents apparently had a better response to training (Fig. 6).

## Discussion

Learning video surgery techniques at the beginning of a surgeon’s career is challenging since such skills are not natural and must be acquired through consistent training [18]. To assist in this process and provide an alternative learning tool, the course was designed in such a way that the practical training exercises were carried out with accessible materials, creating conditions that can be easily reproduced in any environment during medical residency and reduce the inherent costs of the learning curve [5, 12, 19]. The basic training structure was a handcrafted wooden box, with a tutorial provided for its preparation being offered to interested students at the end of the course (Fig. 1). The camera used to view the exercises was the participants’ own cellular phone or tablet. The use of these inexpensive readily available materials may contribute to expand the application of these video-surgery courses, increasing the training opportunities of young surgeons and, hopefully, improving their surgical abilities.

Despite not providing the acquisition of all the skills necessary for the practice of video surgery, short-term training appears to be effective in increasing an individual’s basic skills, resulting in reduced surgical training costs during the learning curve, as well as encouraging independent practice of video surgery [13, 19–21].

Assisted training, with individual guidance by a supervising professor, proved to be superior to self-taught training [13, 17]. The presence of a professor appears to be the factor with the greatest impact on improving skill acquisition in video surgery [22]. In the present course, the students were supervised by one or more instructors, with individual comments on the participants’ performance during and after the course, explaining points of improvement by comparing pre- and post-training (knot quality, ergonomics, camera skill, movement smoothness).

The best way to evaluate the development of surgical skills among residents is still a matter of debate. A recent literature revision states that simulation appears to be an effective and good method for assessing not only technical skills but also judgment, teamwork, and cognitive skills [14]. Error rate reduction when performing exercises in a simulator box has been widely used for evaluating the effectiveness of laparoscopic training [13, 23]. The time taken to perform a given laparoscopic exercise before and after training has also been used and it appears to be easier to control [9, 13, 23]. In the present study, an improvement in the time taken to perform the exercises could be observed after participation in the short-term theoretical-practical course offered during the study. When comparing all participants’ results, it was observed that, after the short-term practical training, there was an increase in the number of individuals who were able to perform the exercise within the maximum time limit of 5 minutes, as well as a significant reduction in the time needed to execute a laparoscopic stitch, indicating the course’s effectiveness in improving participants’ skills. One of the limitations of this study is the absence of a control group without the training phase. Although it could be argued that the simple repetition of the test exercise could improve the time taken to complete the second task, this control group was not included at this moment, because our aim was simply to investigate if this low-cost training method was feasible and effective. The importance of repeated training is widely accepted and the test exercises were not, in this investigation, considered as part of the training.

Subjectively, after the training stage, it was observed that the residents were able to apply what was learned in the course to facilitate the completion of knots. They increased the use of their non-dominant hand to support the structure and assist in movements, employed smoother and more elegant movements, and improved the quality of the knots (fewer “false knots”); also, there was an improvement in the ability to handle the camera (understanding what their fellow surgeon needs to see).

On the other hand, that there was very little improvement in ergonomics, even with the frequent tips given on posture during practice. This aspect could be explained by the excessive concern and attention directed towards the exercise, associated with the difficulties in performing video surgery at this point in the learning curve. Nevertheless, we suggest that ergonomics be taught, corrected and practiced continuously, from the beginning of the surgeon’s training, considering its importance in the long-term quality of life of professionals in the area.

Recent publications have attempted to correlate the playing of video games with greater skill in video surgery procedures [8, 24, 25]. In the present study participants who had video game experience demonstrated a better performance in the pre-training exercise, with a reduction in the time taken to perform the stitch (*p* = 0.0116). This may mean that participants with video game experience may have better eye-hand coordination and find it easier to handle laparoscopic instruments and to deal with two-dimensional vision. This data corroborates previous studies, which correlated surgeons’ previous video game experience with video surgery skills, highlighting the spatial awareness needed for both activities, provided by the complex interaction between the visual and cognitive systems [8, 24–26]. However, this correlation still needs further investigation.

Other factors possibly associated with surgical skills – such as age, gender and area of interest – have also been investigated with varying results [6]. Our results point to a better post-course performance amongst female residents. It is important to note that this is a secondary outcome and that the subdivision into genders also resulted in a sample size reduction of each subgroup, thus reducing the test’s power (approximately 50%). Therefore, in the present settings, these results should be taken carefully.

Nevertheless, investigating a possible gender difference regarding the development of surgical skills has been a frequent subject of study. Overall, published data suggest that male individuals perform better than females after graduation. Amongst surgical residents, this difference is not apparent anymore, possibly associated with the increased interest of all individuals who decide to pursue a surgical speciality. Female individuals, on the other hand, seem to have a better response to assisted individual training, which is corroborated by the apparent improved response amongst female participants to the training offered in the present study. Thus, different advantages seem to be associated with each gender, resulting in similar overall performance among surgeons of both genders [6, 26–28].

Participants’ satisfaction with the course was high, with all 36 participants stating that they would recommend the course “a lot” or “quite a lot” to a colleague also in General Surgery training. When the activities were completed, many showed interest in continuing practising in classes both during the curricular year and at home.

Finally, it is believed that the main contribution of this study is to show that short training courses like this one, performed with low-cost, readily available materials, can be effective and also easily reproduced in any institution that has a surgical residency program, helping to spread the teaching of laparoscopic skills.

## Conclusion

The data collected in this study allow to conclude that the short-term theoretical-practical course using inexpensive handcrafted materials was effective, resulting in reduction in the time taken to perform the proposed laparoscopic exercise.

The data also suggest that individuals with previous video game experience displayed a better pre-training performance, and that female participants had better results (shorter times) in performing the post-training exercise.

Further studies should be implemented to investigate the different factors that may influence learning ability and surgical performance in minimally invasive procedures.

## Data Availability

The datasets used and/or analysed during the current study are available from the corresponding author on reasonable request.
